# In‐hospital mortality as the side effect of missed care

**DOI:** 10.1111/jonm.12965

**Published:** 2020-04-02

**Authors:** Beata Wieczorek‐Wojcik, Aleksandra Gaworska‐Krzemińska, Aleksander J. Owczarek, Dorota Kilańska

**Affiliations:** ^1^ Department of Nursing Institute of Health Sciences Pomeranian University in Słupsk Słupsk Poland; ^2^ Faculty of Health Sciences Institute of Nursing and Midwifery Medical University of Gdansk Gdansk Poland; ^3^ Department of Instrumental Analysis Faculty of Pharmaceutical Sciences in Sosnowiec Medical University of Silesia Katowice Poland; ^4^ Department of Health System Development Medical University of Lodz Department of Coordinated Care Medical University of Lodz Lodz Poland

## Abstract

**Aim:**

The aim of the study was to assess the influence of the number of hours of daily nursing care for NHPPD in medical departments on missed care and the correlation between NHPPD and in‐hospital mortality.

**Background:**

Patient mortality can be a consequence of missed care as it correlates with the nurse–patient ratio. One of the methods to measure missed care is the Nursing Hours per Patient Day rating.

**Methods:**

The study sample included 44,809 patients including 971 deaths in 8 wards. The influence of nursing hours, nursing education, and the percentage of patients' classification on in‐hospital mortality were evaluated with backward stepwise linear regression.

**Results:**

One hour added to the average NHPPD in medical departments was related to a decrease in mortality rate by 6.8 per 1,000 patient days and a lower chance for the emergence of unplanned death by 36%.

**Conclusions:**

The number of NHPPD and the percentage of professional nurses are factors influencing missed care and in‐hospital mortality.

**Implications for Nursing Management:**

The severe consequences of missed care, that is mortality, and the correlation between in‐hospital mortality, nursing education and nursing–patient ratio, which are indicators of care quality, are arguments for maintaining adequate staffing levels to avoid missed care.

## 1 INTRODUCTION

Missed nursing care is a global concern for nurses and nurse administrators and its specificities may vary depending on the cultural context of a country. Investigating the relation between relying on delegation and missed nursing care might help in designing strategies for nurses to minimize missed care and enhance the quality of services (Sager & AbuAlRub, 2018). An analysis of the impact of environmental factors revealed that nurses failed to deliver a significant amount of necessary patient care (10%–27%). Inadequate staffing and inadequate resources were the environmental factors most strongly associated with missed nursing care events (Hessels, Flynn, Cimiotti, Cadmus, & Gershon, 2015).

According to Kalisch & Xie, missed nursing care is substantial and similar levels are found in a number of hospitals. The reasons for missed nursing care in hospitals include staffing resources, material resources and communication issues. The higher the staffing levels, the fewer occurrences of missed nursing care. Missed nursing care predicts adverse events (i.e. falls, pressure ulcers, new infections and patient mortality) (Kalisch & Xie, 2014).

The impact of nurse staffing levels and nurses' education on patient mortality has been researched in several countries (Audet, Bourgault, & Rochefort, 2018; Haegdorens, Van Bogaert, De Meester, & Monsieurs, 2019), unfortunately not in Poland. It has been proven that increasing the nursing ratio and the percentage of qualified nurses reduces in‐hospital mortality (Kane, Shamliyan, Mueller, Duval, & Wilt, 2007b). Low nurse staffing levels are significantly associated with a higher incidence of missed care, which is a more direct indicator of nurse staffing adequacy. Maintaining adequate nurse staffing is instrumental in preventing missed care and thus preventing or delaying in‐hospital mortality (Griffiths et al., 2018). Hospital mortality within 30 days after admission is an effective indicator of the quality of medical services. Nevertheless, according to the Eurobarometer, 28% of Polish patients experience complications as a result of contacting the health care system and not related directly to their illness (Series, 2012). The Polish Accreditation Commission emphasized the necessity of analysing in‐hospital mortality and evaluating which deaths could have been avoided with improved safety of care (National Centre for Quality Assessment in Health Care in Cracow, 2009).

What the results of international studies also indicate is that the number of nurses' working hours and their qualifications influence the occurrence of adverse events, complications and deaths. The RN4CAST project confirmed a relationship between nurses' work overload and in‐hospital mortality on an international level and offered research evidence to the decision‐makers in Europe responsible for optimizing safety and costs of care and avoid missed care. Countries participating in the project, as well as the European Commission, received information on effective methods of influencing the allocation of funds within the health care system, and a long‐term policy concerning medical staff. These results have not been confirmed in Polish studies but it can be assumed that similar dependencies are present in Poland, too. The RN4CAST researchers recommend in‐depth European longitudinal studies, in particular in the countries where such dependencies have not been identified yet (Aiken et al., 2014).

High‐quality data enabling an analysis of adverse events incidence on a national level are not gathered in Poland. A relation between the nurse staffing level and nursing degree levels versus the results of patients' treatment has not been found so far, as shown in the RN4CAST study. With regard to the implementation of standards of minimal employment of nurses per hospital bed in Poland since January 2019, that is 0.6 in medical departments and 0.7 in surgical ones, the trend is to replace professional nurses with medical carers, whose number is insufficient and they only work in some hospitals. In most hospital wards, nurses have to work on their own without the support from medical carers. Additionally, there are no national recommendations concerning optimal nurse staffing level based on the indicators of care safety, which could help Nursing Managers to eliminate the problem of missed care.

Health care facility managers are in charge of patients' safety and a number of staff members taking care of them. Calculations of nurse care needs are not always reflected in reality. Decision‐makers do not consider the implementation of good practices. They think that the results of such projects as the RN4CAST cannot be applied in Poland due to different epidemiological context.

In Poland, there are two different nurse education levels:

1. Nurses with higher education—the bachelor's degree nurse (European Qualification Framework, EQF level 6, BA) and master's degree nurse (EQF level 7, MA).
2. Certified nurses who graduated from nursing high schools (EQF level 4/5). These schools were in operation until 2002.

The nursing staff is still perceived as a cost, not an investment because no one calculates the costs of adverse events generated by an inefficient nursing staff. At the same time, there is an ongoing political debate in Poland on reinstating nursing education on a secondary level, which results from an insufficient number of nurses. Therefore, evidence is required confirming the relationship between nursing education and patients' outcomes, which correlate with missed care.

The studies conducted by the authors are also justified by the conclusions from the RN4CAST cross‐sectional study which demonstrated that each additional patient assigned to a nurse was related to an increased probability of in‐hospital mortality within 30 days after admission by 7% (Aiken et al., 2014). Due to the limitations of a cross‐sectional study in cause and effect reasoning, the need for local, longitudinal studies was suggested to confirm these dependencies.

The need for such studies is also confirmed in a systematic review of studies in the field (Audet et al., 2018) which indicate that while evidence suggests that higher nurse education is associated with lower risks of mortality and failure to rescue, longitudinal studies are needed to better ascertain these associations and determine the specific thresholds that minimize risks.

Identification of causes and indicators of missed care is one of the most important elements. It allows managers to properly plan their nursing needs. The study conducted in Poland meets these criteria.

The aim of the study was to assess the influence of the number of nursing hours per patient days (NHPPD) in surgical and medical wards on missed care and the correlation between NHPPD and in‐hospital mortality.

## 2 METHODS

This was a retrospective observational longitudinal study. The study consisted in an analysis of patients' deaths, basing on statistical charts, and nursing staff data in regard to NHPPD and classification of patients (according to the Polish regulations). All patients in 8 wards (4 medical and 4 surgical wards) in the study period (3 years) were enroled in the study. The study population was 44,809 adults, including 971 deaths. The study included all nurses working in those wards—528 nursing staff positions ‐ and a total of 757,438 nursing hours. At that time, the hospital did not employ medical carers.

Patients' demographic data and list of deaths were collected from electronic patient records—EPR, CLININET and SERRUM database. Nursing staff data were collected in an Excel sheet with additional calculating functions developed by the authors. The department nursing staff data set was monitored daily. The NHPPD number is a mean value obtained after dividing the actual nursing hours per day by the number of hospitalized patients.

The term *professional nurse* refers to fully qualified professional nurses (the highest academic qualification was a bachelor's degree or higher).

The number of professional nurses at each shift was also monitored. On this basis, the percentage of professional nurses in individual quarters and years was calculated. Data concerning the number of ‘patient days’ for particular patients' classification (patient classification system—PCS) were also registered on a daily basis. The controlled clinical study covering three consecutive years (January 2012–December 2014) included 12 quarters of three consecutive months of the analysed period. The analysis of deaths included avoidable in‐hospital mortality, according to the guidelines of the National Centre for Quality Assessment in Health Care in Cracow (NCQAHC), in the group of patients aged 18+, from surgical and medical wards, who stayed in a hospital longer than 24 hr (971 registered in the analysed period). According to the NCQAHC guidelines, so‐called unplanned deaths are defined as in‐hospital mortality, excluding patients hospitalized initially in the intensive care ward or being in a terminal status (Wojtyniak & Goryński, 2018). Patients were classified as per ICD‐9 and ICD‐10 codes. The mortality rate was calculated assuming that in‐hospital mortality is the number of deaths due to a given disease in relation to the number of cases of this disease at the same time (Bzdęga & Gębska‐Kuczerowska, 2010)

Next, a relation between NHPPD rate in surgical and medical wards, nurses' education and hospital mortality due to unplanned hospital deaths and the PCS (Polish adaptation by Ksykiewicz‐Dorota) was estimated. The PCS independence level was evaluated in the following aspects: physical activity, hygiene, nutrition, excretion, life parameters, treatment and health education, and social support.

The number of nursing hours, the percentage of professional nurses and the percentage of PCS = III patients between wards were compared with the Kruskal–Wallis nonparametric variance analysis. The nonparametric Mann–Whitney *U* test was used for the comparison of surgical and medical wards. The influence of nurses' hour rate, nurses' education and the percentage of PCS = III patients on hospital mortality were estimated with a backward stepwise linear regression. Variables with significance level *p* < .05 were considered as statistically significant parameters (Statistica 10.0).

## 3 RESULTS

Data were collected for the deaths of 971 patients within 30 days after admission or later (single events). In‐hospital mortality amounted to 2.2% for this group, and in‐hospital mortality rate per 1,000 patient days = 4.46. The higher in‐hospital mortality rate was found in medical wards (6.23) and a lower one in surgical wards (1.58) (Table 1).

**Table 1:** Mortality in total including surgery and medical wards in the years 2012–2014

Hospital		2012				2013				2014				Total
Wards	*N*	I	II	III	IV	I	II	III	IV	I	II	III	IV	
Surgical wards	Events	0	0	0	15	15	19	16	14	14	15	13	10	131
	Patient days	6,630	6,009	5,759	5,891	7,350	8,344	8,246	6,750	7,373	7,095	6,509	7,057	83,013
	Incidence	0.00	0.00	0.00	2.55	2.04	2.28	1.94	2.07	1.90	2.11	2.00	1.42	1.58
Medical wards	Events	0	0	0	60	104	98	79	89	108	85	110	107	840
	Patient days	11,534	11,142	10,163	10,714	12,535	12,642	11,794	10,767	11,135	10,255	10,995	11,117	134,793
	Incidence	0.00	0.00	0.00	5.60	8.30	7.75	6.70	8.27	9.70	8.29	10.00	9.62	6.23
Total	Events	0	0	0	75	119	117	95	103	122	100	123	117	971
	Patient days	18,164	17,151	15,922	16,605	19,885	20,986	20,040	17,517	18,508	17,350	17,504	18,174	217,806
	Incidence	0.00	0.00	0.00	4.52	5.98	5.58	4.74	5.88	6.59	5.76	7.03	6.44	4.46

Note

Incidence calculated per 1,000 patient days.

NHPPD rate amounted to 4.2 hr on average. The highest NHPPD rate was noticed in surgical wards (4.79) and the lower by 1.15 hr in medical wards (Table 2).

**Table 2:** Nursing hours per patient day (NHPPD) in individual wards in the years 2012–2014

Wards	2012				2013				2014			
	I	II	III	IV	I	II	III	IV	I	II	III	IV
General surgery	3.84 ± 0.61	4.38 ± 1.20	4.55 ± 1.12	4.34 ± 1.19	3.96 ± 1.02	3.53 ± 1.14	3.96 ± 1.29	4.01 ± 1.46	3.89 ± 0.80	3.87 ± 0.82	4.39 ± 1.25	3.87 ± 0.80
Laryngology	5.82 ± 3.87	4.99 ± 3.83	5.68 ± 3.27	5.96 ± 5.12	5.35 ± 5.49	4.56 ± 3.68	4.99 ± 4.45	6.61 ± 6.23	5.38 ± 3.75	8.50 ± 8.62	7.39 ± 6.10	5.19 ± 4.11
Urology	5.30 ± 1.97	5.03 ± 1.84	7.91 ± 6.61	5.91 ± 2.69	4.89 ± 3.97	4.16 ± 2.91	4.60 ± 2.82	6.60 ± 6.10	5.87 ± 6.42	5.34 ± 4.35	6.19 ± 8.38	7.98 ± 9.28
Orthopedics and traumatology	4.94 ± 1.43	4.84 ± 1.69	3.98 ± 0.93	5.19 ± 1.18	3.91 ± 1.19	3.28 ± 0.86	3.02 ± 1.18	3.76 ± 0.71	3.74 ± 1.21	3.35 ± 1.08	3.53 ± 0.75	3.3 ± 0.94
Surgical wards	4.98 ± 2.41	4.81 ± 2.37	5.31 ± 2.63	5.35 ± 3.07	4.52 ± 3.5	3.88 ± 2.49	4.14 ± 2.86	5.15 ± 4.32	4.53 ± 2.89	5.27 ± 5.26	5.05 ± 4.02	4.71 ± 3.67
Internal diseases	3.59 ± 0.51	3.50 ± 0.58	4.08 ± 0.74	4.23 ± 0.78	3.91 ± 0.45	4.31 ± 0.88	4.44 ± 0.57	3.90 ± 0.54	3.97 ± 0.48	4.14 ± 0.61	3.97 ± 0.58	3.65 ± 0.35
Cardiology	3.41 ± 0.46	3.74 ± 1.00	3.72 ± 0.76	3.57 ± 0.72	3.46 ± 0.54	3.44 ± 0.49	3.75 ± 0.68	3.74 ± 0.70	3.37 ± 0.55	3.92 ± 0.72	3.57 ± 0.69	4.28 ± 1.71
Neurology	5.61 ± 1.08	4.61 ± 0.99	5.04 ± 1.15	5.36 ± 1.28	4.46 ± 0.77	4.20 ± 0.77	4.10 ± 0.6	4.42 ± 0.87	4.90 ± 1.05	4.38 ± 0.67	4.19 ± 0.91	4.37 ± 0.78
Diseases of the lungs	2.60 ± 0.56	2.96 ± 1.03	4.15 ± 1.30	3.18 ± 0.74	2.17 ± 0.37	2.00 ± 0.26	2.24 ± 0.49	4.10 ± 2.06	3.23 ± 0.87	3.27 ± 0.66	3.51 ± 0.92	3.22 ± 0.97
Medical wards	3.67 ± 1.34	3.57 ± 1.12	4.01 ± 1.19	3.86 ± 1.23	3.29 ± 0.99	3.2 ± 0.97	3.33 ± 0.89	4.04 ± 2.22	3.62 ± 1.07	3.66 ± 0.82	3.56 ± 0.88	3.67 ± 1.33
Total	4.32 ± 2.06	4.19 ± 1.95	4.66 ± 2.14	4.60 ± 2.45	3.91 ± 2.64	3.54 ± 1.92	3.74 ± 2.16	4.60 ± 3.48	4.07 ± 2.22	4.47 ± 3.84	4.3 ± 2.98	4.19 ± 2.80

The percentage of professional nurses amounted to 32.2% for medical wards and 42.3% for surgical ones. A total of 971 patients classified per PCS‐III were treated in an internal ward, cardiology and neurology, as well as a general surgical ward. The study compared the level of nursing staff (NHPPD), nursing education (NE) and profile of hospitalized patients (percentage of PCS = III—CP patients) versus unplanned patients' deaths (Table 3).

**Table 3:** Backward stepwise regression (*β* (*SE*)/100) of the impact of nursing hours per patient day (NHPPD), nurses' education (HE—higher education) and the percentage of severely ill patients (SIP) on the type and frequency of individual adverse events

Deaths	SIP	NHPPD	HE
Surgical wards	–	–	−0.3250 (0.0448)$
Medical wards	–	−6.8171 (2.3547)*	−0.7529 (0.1385)$

* *p* < .05.

^$^ *p* < .001

It was demonstrated that as for medical wards, one additional hour of NHPPD prevents missed care and reduces the number of deaths by 6.8 per 1,000 patient days.

In‐hospital mortality rate decreases along with an increase in nursing care rate; the more professional nurses, the fewer deaths at surgical and medical wards. An increase in the nurses' rate by 10% causes a decrease of in‐hospital mortality by 3.25 per 1,000 patient days and by 7.53 per 1,000 patient days, respectively.

The results of the analysis demonstrated that increase in an average NHPPD number in medical wards from 3.64 to 4.64 was related to a decreased chance of an unplanned death event by 36% (OR = 0.64; (OR 95% CI: 0.57–0.71; *p* < .01). This dependence is statistically significant. Therefore, the 4.64 NHPPD rate should be considered as optimal in medical wards, which can minimize the risk of missed care. In surgical wards, the actual average NHPPD 4.79 rate demonstrates no relation to unplanned in‐hospital mortality; therefore, it can be considered as an optimal rate to be maintained in practice and decrease the level of missed care. An increase in percentage of professional nurses from 32.2% to 42.2% in medical wards decreases the chance for an unplanned death by 7%; however, this dependence is statistically insignificant (OR = 0.93; 95% CI: 0.83–1.12; *p* = 1.08). An increase in the percentage of professional nurses from 42.3% to 52.3% in surgical wards decreases the risk of death by 17% (OR = 0.83; 95% CI: 0.64–1.07; *p* = 1.20). Therefore, the results of this study demonstrate that these incidents of hospital missed could be improved by increasing the level of nursing care.

## 4 DISCUSSION

This is the first Polish study with a complex analysis of dependence between the number of NHPPD (measured systematically during 3 years, on a daily basis), the percentage of professional nurses and the in‐hospital mortality to show the problem of missed care. It enables the comparison of the results with analogous findings of international studies. Previous research evidence included mostly results of cross‐sectional studies, which are limited in cause and effect reasoning.

Avoidable in‐hospital deaths are correlated with missed care, which includes the failure to identify a patient's worsening condition early and respond adequately (National Patient Safety Agency (Great Britain) 2007).

The Agency for Healthcare Research and Quality indicates that in the United States in the years 2010–2013, thanks to actions towards improvement of care quality, 1.3 million of patients avoided adverse events, what enabled prevention of 50,000 deaths (Agency for Healthcare Research & Quality, 2019).

Literature review enables the identification of numerous studies evaluating dependence between the level of nursing staff and the in‐hospital mortality. The dependence between NHPPD and in‐hospital mortality was proved in the *Needleman* study, where the risk of death at each shift amounted to 1,02 (95% CI: 1,01–1,03, *p* < .001) (Needleman et al., 2011). These results were confirmed by McHugh in 2013, demonstrating a statistically significant dependence between nursing missed care rates and 30‐day in‐hospital mortality in a Magnet Hospital. Lower in‐hospital mortality was significantly related to nursing missed care (McHugh et al., 2013). A meta‐analysis of 2007, on a sample of 90 studies on AHRQ data, confirmed that increased nursing staff is related to a decrease in adjusted odds ratio for in‐hospital mortality and missed care. An increase by each one nursing regular post per patient days was effected in a decrease of in‐hospital mortality in medical and surgical wards. An increase in a total value of NHPPD was related to a decrease of in‐hospital mortality, and each additional hour of NHPPD decreased in‐hospital mortality by 1.98% (Kane, Shamliyan, Mueller, Duval, & Wilt, 2007a). The study by Blegen, Goode, Spetz, Vaughn, and Park (2011) run in 872 surgical and medical wards demonstrated that high NHPPD rate is related to decrease of in‐hospital mortality (*β* = −0.087; 95% Cl: −0.15 to 0.02 *p* < .05) (Blegen et al., 2011). Aiken, Sochalski, and Lake (1997) also proved that nursing/patient or NHPPD rates are significantly correlated with in‐hospital mortality and missed care. In hospitals with higher proportions of nurses educated at the undergraduate level or higher, surgical patients experienced lower mortality and failure‐to‐rescue rates (Aiken, Clarke, Cheung, Sloane, & Silber, 2003). An increase in nursing load by an additional patient under his/her care causes a 7% (OR‐1.07; 95% CI 1.03–1.12) increase in the risk of in‐hospital mortality (Aiken, Clarke, Sloane, Sochalski, & Silber, 2002). The study of Swiss hospitals demonstrated also that in the hospitals with higher patient/nurse rates (10:1), the risk of in‐hospital mortality was higher by 37% (OR 1.37, 95% CI:1.24–1.52) (Schubert, Clarke, Aiken, & de Geest, 2012).

Within this project, it was demonstrated that an additional 1 NHPPD decreases the number of in‐hospital mortality by 6.8 per 1,000 patient days in medical wards, and the risk for in‐hospital mortality decreases by 36%. The dependence between nursing staff, missed care and the higher in‐hospital mortality rate manifests itself in neglecting important elements of care, such as monitoring vital signs and delayed notification of worsened patient's status in consequence. Such neglects can bring significant consequences to the patients, as they increase a risk of serious clinical problems and/or inadequate treatment of complications. The reasons for higher in‐hospital mortality rate may include decreased quantity or quality of nursing care offered to the patients and missed care.

Literature review reveals that a higher proportion of professional nurses is related to decreased in‐hospital mortality rate and missed care (Audet et al., 2018; Estabrooks, Midodzi, Cummings, Ricker, & Giovannetti, 2005; Haegdorens et al., 2019; Tourangeau et al., 2007). Meta‐analysis of data for the years 1990–2006 demonstrated that each additional professional nurse post decreases mortality by 9%–16% (Kane et al., 2007a). An analysis of 118,940 patients of American hospitals proved lower in‐hospital mortality in hospitals with higher proportion of professional nurses, what is related with higher quality of care, and observed in large hospitals mostly (Person, Allison, Kiefe, & Weaver, 2004). The RN4CAST project demonstrated that an increase in the number of professional nurses versus all employed nurses by 10% was related with decrease in a probability of in‐hospital mortality within 30 days after admission by 7% (OR = 0.94, 95% CI: 0.90–0.97; *p* < .002) (Aiken et al., 2014). The same was proved in the study performed in the Ceynowa Hospital in Wejherowo which revealed that the in‐hospital mortality rate decreased along with an increase in a nursing care rate, the more professional nurses—the lower amount of deaths in surgical and medical wards. A 10% increase in the percentage of nurses results in a decrease of in‐hospital mortality by 3.25 per 1,000 patient days and 7.53 per 1,000 patient days, respectively. When a percentage of professional nurses was higher by 10%, a chance for in‐hospitality mortality decreased by 8% in medical and by 17% in surgical departments, which was important for preventing missed care. However, these results should be analysed with caution due to lack of statistical significance, caused most likely by a small number of adverse events in the analysed period.

In‐hospital mortality in Poland, according to the *Report on the health situation of the population in Poland* offered by the National Institute of Public Health, amounted to 2.3% in 2003 (patients hospitalized due to all reasons in total). It decreased by 2.1% in 2006 and amounted to 1.9% in 2016 (Wojtyniak & Goryński, 2018). According to Polish Ministry of Health, the number of in‐hospital mortality due to surgical reasons amounted to 4,527 (which makes 0.5% of the number of hospitalizations), and number of in‐hospital mortality due to non‐surgical reasons amounted to 7,729 (0.9% in relation to the number of hospitalizations (Kulig & Legutko, 2008). In the American study, the in‐hospital mortality rate amounted to 3.1% in 2012 (Glance et al., 2012). In Great Britain, in the years 2008–2009 the in‐hospital mortality rate per 1,000 patient days in emergency hospitals, basing on the sample of 27,582 deaths within 30 days after admission, among 4 133 346 patients hospitalized in surgical wards, amounted to 6.7 (Aylin, Alexandrescu, Jen, Mayer, & Bottle, 2013). In the project presented here, the in‐hospital mortality rate, in general, amounted to 4.46 per 1,000 patient days. This makes 2.2% of deaths in relation to all hospitalized patients and is comparable with the results of the analysed studies. The in‐hospital mortality rate in surgical wards amounted to 1.58 per 1,000 patient days and was significantly lower than in the British study, and in medical wards, it amounted to 6.23 per 1,000 patient days and was comparable to the one in the British project. The highest in‐hospital mortality rate was recorded in the internal ward—11.18 per 1,000 patient days.

The results of international studies indicate clearly that activities for patients' safety cannot be considered disregarding nursing work, as a low level of nursing staff results in a critical increase in patients' morbidity and in‐hospital mortality (International Council of Nurses, 2006). Increased employment can improve patients care through a decrease in unnecessary deaths (Stone et al., 2007). The results of the study by Aiken et al. (2014) conducted in 9 countries (Belgium, Great Britain, Finland, Ireland, the Netherlands, Norway, Spain, Sweden and Switzerland) indicate that a secure level of nursing staff in a hospital can contribute to decreasing in‐hospital mortality in surgical wards. An adequate level of nursing staff and the proportion of professional nurses is inversely proportional to 30‐day in‐hospital mortality in emergency hospitals (Tourangeau et al., 2007). Research evidence indicates clearly that employment of nurses, even though generating costs, is actually rewarding due to a financial burden of treatment of adverse events (Aiken, 2008).

As experienced by other countries, individual studies concerning dependences between the level of nursing staff and adverse events contributed significantly to a wider discussion and actions for improvement of patients' safety. Evidence for such a relationship has not been found in Polish literature. Therefore, the authors decided to conduct the study on a local level. The study proved the necessity of maintaining the current model of nursing education in Poland (higher education). The conclusions from the RN4CAST project have been confirmed. The results indicating relationship between the level of nursing staff and decreased risk of in‐hospital mortality are inconvenient for the Polish health care system in the current financial situation.

## 5 LIMITATIONS

This is the first study in Poland estimating the influence of nursing staff level on in‐hospital mortality, concerning avoidable deaths and at the same time (due to its limitations) realized in one hospital only. This is due to a difficulty in obtaining the agreement of hospitals' managements, despite many attempts of realization of the study on a wider scale. Management of one hospital expressed some interest but the chief nurse did not implement adequate tools. Tools enabling the collection of information indispensable for further analyses are not commonly used in Poland. Access to databases of adverse events and nursing staff is currently impossible. A multilateral study would be possible after the introduction of an obligatory register of adverse events and development of other e‐registers on a national scale.

## 6 CONCLUSION

The findings show that there is a causal relationship between the nurse staffing level and nursing education versus in‐hospital mortality, which is in correlation with missed care. It means that evidence‐based investments in nursing will bring measurable benefits for the society and the payer.

National comparative studies on patients' safety indicators in different medical units, including those accredited as offering higher quality care, should be continued. Studies should be conducted in a large number of hospitals to follow up on the proven dependencies between missed care and in‐hospital mortality, as well as current and expected shortages of nurses. Polish hospitals should apply comparative analyses to evaluate the level of missed care, as well as refer to research evidence evaluating relationship between missed care and patient outcomes.

The results of the study confirm those from the RN4CAST and earlier international projects and point to the chance to reduce missed care in Polish hospitals, and a decrease of in‐hospital mortality. Besides improved safety, investments in nursing can bring some savings for the health care system, which struggle with huge financial problems. Managers should seek for the maintenance of average NHPPD in surgical departments on 4.79 level and 4.64 in medical ones. The percentage of professional nurses should be maintained on a minimal level of 42.2% in medical departments and 52.3% on surgical ones. The results should be verified in future studies in other Polish hospitals.

## 7 IMPLICATIONS FOR NURSING MANAGEMENT

The severe consequences of missed care, that is mortality, and the correlation between in‐hospital mortality, nursing education and nursing–patient ratio, which are indicators of care quality, are arguments for maintaining adequate staffing levels to avoid missed care. If nursing managers know the impact of these indicators on care, they will be able to optimize care in terms of safety and cost and make decisions in line with EBN and management ethics.

## ETHICAL APPROVAL

The study was approved by the Bioethics Commission at the Medical University of Gdansk, No NKBBN/41/2015, and the hospital management on 20.01.2015.
